# Tears of wine: new insights on an old phenomenon

**DOI:** 10.1038/srep16162

**Published:** 2015-11-09

**Authors:** David C. Venerus, David Nieto Simavilla

**Affiliations:** 1 Department of Chemical and Biological Engineering, Illinois Institute of Technology, Chicago, IL 60616.

## Abstract

Anyone who has enjoyed a glass of wine has undoubtedly noticed the regular pattern of liquid beads that fall along the inside of the glass, or ‘tears of wine.’ The phenomenon is the result of a flow against gravity along the liquid film on the glass, which is induced by an interfacial tension gradient. It is generally accepted that the interfacial tension gradient is due to a composition gradient resulting from the evaporation of ethanol. We re-examine the tears of wine phenomenon and investigate the importance of thermal effects, which previously have been ignored. Using a novel experiment and simple model we find that evaporative cooling contributes significantly to the flow responsible for wine tears, and that this phenomenon occurs primarily because of the thermodynamic behavior of ethanol-water mixtures. Also, the regular pattern of tear formation is identified as a well-known hydrodynamic instability.

Tears of wine, or wine tears, is a well-known phenomenon that can be described as the continuous formation of liquid beads that fall along the inside of a stationary wine glass. In 1855, J. Thomson^1^ identified the driving force for the upwards flow necessary for the continuous formation of wine tears as a gradient in interfacial tension. Some 15 years after Thomson, Marangoni^2^ described this phenomenon, which is commonly associated with his name^3^.

As shown in Fig. 1 ^4^, the film begins at the meniscus. Near the top, the film becomes thicker forming a ridge, which is bound by an essentially stationary contact line where the glass, wine and air phases coexist. With fairly regular spacing, tears form from the ridge and fall along the film towards the meniscus, which will occur for several minutes if the glass remains stationary. The meniscus and ridge are connected by a thin liquid film where there is an upwards flow that is driven by a Marangoni stress. The ability to observe this phenomenon, and some of its quantitative features such as the film shape, tear spacing, and frequency, depend on the type of wine and the surface properties of the wine glass. In other words, the tears of wine phenomena results from a delicate interplay of interfacial and bulk forces.

There has been considerable interest in understanding the tears of wine phenomenon^5^,^6^,^7^,^8^,^9^. Much of this work has focused on the origin of the Marangoni flow, shape of the liquid film, and the instabilities leading to the formation of tears. It is generally accepted that the flow leading to wine tears is due to a composition gradient that results from the evaporation of ethanol, which has a smaller interfacial tension than water. Thermal effects resulting from evaporative cooling have been ignored in all previous studies^5^,^6^,^7^,^8^,^9^.

Bulk flows driven by interfacial forces are ubiquitous in nature and are critical to biological function, materials processing, and engineering. Considerable experimental and theoretical work has focused on film spreading, or wetting, and flow instabilities that are commonly observed in films driven by Marangoni stresses^10^,^11^,^12^,^13^,^14^. In addition to interfacial tension gradients, capillary pressure due to curvature of the interface, and interfacial tension differences at contact lines, can be manipulated to produce astonishing phenomena^15^. Liquid drops moving up inclined surfaces^16^ and super-spreading^17^,^18^ are just two of numerous examples.[Fig f1]

In this study, we investigate tears of wine with the goal of establishing a more comprehensive understanding the mechanisms that lead to the phenomenon. First, we examine the role of thermal effects induced by evaporative cooling, which previously have been ignored, in the Marangoni flow required for wine tear formation. Second, we examine the underlying physics that lead to the highly regular pattern observed in wine tears. To address these questions we use a combination of infrared thermography and classical hydrodynamics. Our analysis shows that evaporative cooling contributes significantly to the flow responsible for wine tears, and that this phenomenon occurs in wine and other spirits because of the thermodynamic behavior of ethanol-water mixtures. We also identify the origin of tear formation as a well-known hydrodynamic instability.

## Hydrodynamic Model

In order to understand the phenomenon of wine tears, we begin with a simple fluid dynamics analysis applicable to a region of the film between falling tears. Consider a liquid film with thickness *δ*(*z*) on an impermeable, solid surface (glass) at an angle *β* with respect to gravity as shown in Fig. 2. The liquid (wine) is modeled as an ethanol-water mixture with ethanol mass fraction 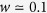 that behaves as a Newtonian fluid with constant density *ρ* and viscosity *η*. We invoke the quasi-steady state approximation, and take the film (neglecting curvature of the glass) to be infinitely wide in the *y*-direction. Hence, the velocity field has the form: *v*_*x*_ = *v*_*x*_(*x*, *z*), *v*_*z*_ = *v*_*z*_(*x*, *z*) and is divergence free:


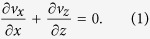


The film between the meniscus and contact line has height *h* ~ 10 mm and characteristic thickness *δ*_0_ ~ 30 *μ*m. Since *δ*_0_/*h* ≪ 1, we assume the lubrication approximation^19^,^20^ holds so that pressure varies only in the *z*-direction. If we further assume inertia can be neglected, the Navier-Stokes equations simplify to the following:


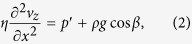


where the prime indicates a derivative along the film: (..)′ = *d*(..)/*dz*, and *g* is the gravitational acceleration. Taking the solid-liquid interface (*x* = 0) to be impermeable and assuming no-slip we can write:


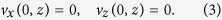


Applying interfacial (jump) balances^21^,^22^ for mass and momentum at the liquid-gas interface (*x* = *δ*), leads to the following boundary conditions:


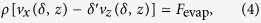



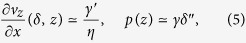


where higher order terms (products of primed quantities) have been neglected. *F*_evap_ is the mass flux into the gas due to evaporation, *γ* is interfacial tension, and *γ*′ is the Marangoni stress. The second boundary condition in eq. (5) gives the capillary pressure due to curvature of the interface.

In the absence of flow, eq. (2) can be combined with the third boundary condition in eq. (5) and integrated to obtain an expression for the film height at equilibrium: *δ*(*h*_eq_) = 0. This well-known solution^23^ is for *β* ≪ 1 given by 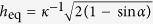, where 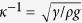 is the capillary length, and *α* is the contact angle. For an ethanol-water mixture at room temperature (*w* = 0.1, 298 K) we find, using *ρ* = 973 kg/m^3^ and *γ* = 55.4 mN/m^24^, the following 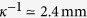. We have measured the contact angle at equilibrium (see Methods) and obtain the value *α* = 14 ± 1°, which gives 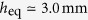. Note that this is roughly three times smaller than the film height observed for wine tears. Several previous studies^7^,^8^,^9^ have addressed spreading phenomena, which leads to larger film heights, in evaporating liquid films. The motion of contact lines is a complex phenomenon^25^; for this analysis we consider the quasi-steady case where the contact line is effectively stationary.

Here we focus on regions of the liquid film away from the meniscus and ridge so that 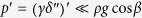
9. Applying this condition, integration of eq. (2) with the boundary conditions in eqs. (3b) and (5a) leads to the following expression:


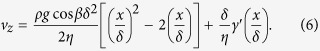


Hence, the velocity along the liquid film is determined by the competition between gravitational and interfacial forces (a sketch of this velocity distribution is shown in Fig. 2). From eq. (6) we can obtain a rough estimate of the interfacial stress required to overcome gravity: *γ*′ > *ρg* cos *βδ*/2 ~ 100 mPa.

Interfacial tension is a thermodynamic property that depends on temperature and composition (ethanol mass fraction): *γ* = *γ*(*T*, *w*). For small variations of temperature and composition, we can write:


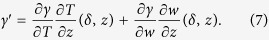


Previous analyses of the tears of wine phenomenon have treated *γ*′ as a parameter and, as noted above, neglected the contribution of thermal effects^7^,^8^,^9^,^10^,^11^,^12^,^13^,^14^.

We now consider the balance equations for ethanol mass and for energy within the liquid film. To keep our analysis simple, we neglect Soret and Dufour effects, the enthalpy of mixing, and viscous dissipation^19^. The ethanol mass fraction *w*(*x*, *z*) is governed by


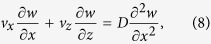


where we have taken the mass diffusivity *D* to be constant. The temperature *T*(*x*, *z*) is governed by the following:


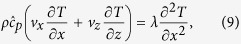


where 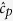 is the specific heat capacity of the liquid, and *λ* is the thermal conductivity, which is taken to be constant. Note that in writing eqs. (8) and (9) we have neglected diffusive transport in the *z*-direction, which is consistent with the scaling used to invoke the lubrication approximation. At the entrance to the film, the temperature and concentration are uniform: *T*(*x*, 0) = *T*_0_; *w*(*x*, 0) = *w*_0_. The expression for velocity along the liquid film eq. (6) is, through eq. (7), coupled to the mass and energy balances in eqs. (8) and (9).

Equations (8) and (9) each require two boundary conditions. Assuming the impermeable solid is a perfect insulator, we have the following boundary conditions at the solid-liquid interface:


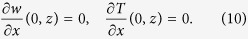


The jump balances for mass and momentum at the liquid-gas interface (*x* = *δ*) imply the excess mass (and momentum) density of the interface is zero. Since interfacial tension is a thermodynamic variable associated with the interface *γ* = *γ*(*T*, *w*), care must be taken in writing species mass and energy balances at the interface^22^. Here, for simplicity, we assume the excess ethanol mass and energy densities can be neglected. Hence, the jump balances for ethanol mass and energy at the interface can, assuming only ethanol evaporates, be written as follows:


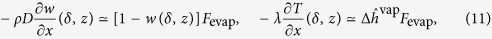


where 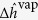 is the specific enthalpy difference between ethanol in vapor and liquid states. In writing the boundary conditions in eqs. (11), we have again neglected higher order terms.

We assume *F*_evap_, the ethanol mass flux at *x* = *δ*, is given by the product of a mass transfer coefficient *k*_g_, and the difference between the gas phase concentrations at the liquid-gas interface and the bulk gas^19^. From equilibrium thermodynamics we know the gas phase concentration of ethanol is related to its concentration in the liquid phase. For ideal mixtures, the relation is linear with a proportionality factor given by the ratio of pure-component vapor pressure to total pressure: *p*^vap^/*p*. Deviations from ideal behavior are taken into account multiplying this ratio by a concentration-dependent activity coefficient 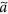. Hence, assuming the ethanol concentration in the air far from the interface is negligible, we can write *F*_evap_ = *k*_g_*φw*(*δ*, *z*) where the factor *φ* is given by 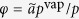. Figure 3 shows that *φ*, where 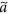 was determined using standard methods^26^, is a strong function of ethanol concentration *w*.

**Figure 1 f1:**
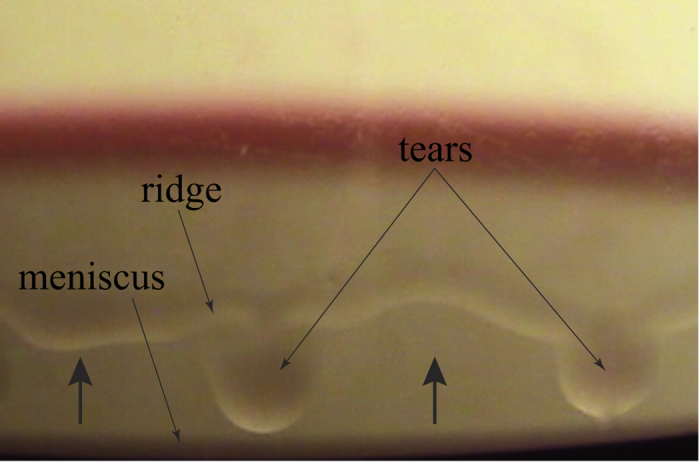
Image of tearing wine film showing regularly spaced tears falling from ridge^4^. Thick arrows indicate upwards flow from meniscus to ridge induced by Marangoni stress. The image shows an area of approximately 18 × 30 mm.

We are interested in regions of the film where there is a net upwards flow (in the *z*-direction) - for example, the thick black arrows in Fig. 1. To proceed, it is convenient to introduce the average across the film thickness: 
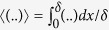
, so that from eq. (6), substituting eq. (7), we obtain


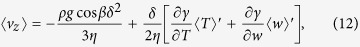


where we have used the approximations 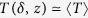 and 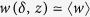. A necessary condition for continuous tearing is that there is a net flow up the liquid film, or 〈*v*_*z*_〉 > 0.

The integrated form of the mass balance eq. (1) for the liquid film, using the boundary conditions in eqs. (3a) and (4), takes the form:


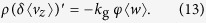


Similarly, using eqs. (4), (10a) and (11a), the ethanol mass balance for the liquid film eq. (8) can be written as


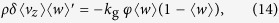


where we have used eq. (13) and the approximation 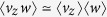. Equation (14) expresses the balance between convective mass transport along the film and the evaporative mass flux to the gas phase. Finally, using eqs. (4), (10b) and (11b), the energy balance for the liquid film eq. (9) takes the form


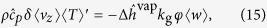


where we have used the approximation 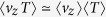. Equation (15) expresses the balance between convective energy transport along the film and the energy required for the evaporation of ethanol.

Equations (12)–(15), 27, 28, 30 are a coupled system of ordinary differential equations that govern film thickness, average velocity, ethanol concentration and temperature within the film. These equations can be integrated from reference values *δ*_0_, 〈*v*_*z*_〉_0_, *w*_0_ and *T*_0_.

## Results

To examine the contribution of the temperature gradient *T*′ to the Marangoni stress, we have used infrared thermography to measure the temperature distribution in a tearing wine film. A typical infrared image taken from a video (see Supplementary Information) is presented in Fig. 4. This image shows two cooler regions with the shape of falling tears between which there is a region with temperature gradient in the *z*-direction. We interpret the region between the falling tears as the region where Marangoni flow occurs. Figure 5 shows the temperature profile along the red vertical line in Fig. 4. From this figure we find for the temperature gradient *T*′ ~ −100 K/m. To estimate the concentration gradient we combine eqs. (14) and (15), which gives:


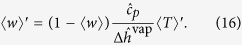


This leads to *w*′ ~ −0.5 1/m, which is consistent with measurements reported elsewhere^9^. For an ethanol-water mixture at room temperature (*w* = 0.1, 298 K), we have 
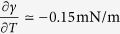
-K and 
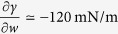
24, so that 
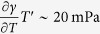
, and 
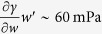
. *Hence, the contributions of composition and temperature gradients to the Marangoni stress have comparable magnitudes*.

For the case just considered, the change in ethanol concentration along the film is less than one percent 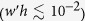. Based on this, we seek an approximate solution to eqs (12)–(15), 27, 28, 30 that is valid for small changes in *w*. First, we multiply eq. (12) by 〈*v*_*z*_〉 and combine with eqs. (14) and (15). Setting *δ* → *δ*_0_, 〈*w*〉 → *w*_0_ and 〈*v*_*z*_〉 → 〈*v*_*z*_〉_0_, we obtain a quadratic equation for 〈*v*_*z*_〉_0_, which has two (real) solutions. Taking the positive solution, we find


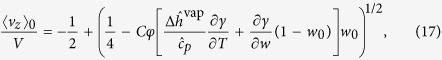


where 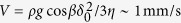 is a characteristic velocity, and *C* = *k*_g_/(2*ρηV*^2^). Note that the derivatives of interfacial tension are evaluated at *w*_0_. The expression in eq. (17) allows for an examination of a necessary condition for wine tears to be observed: 〈*v*_*z*_〉_0_ > 0, and its dependence on the thermodynamic properties of ethanol-water mixtures.

It should be noted that ethanol-water mixtures display moderate deviations from ideal solution behavior. Setting 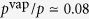, we see from Fig. 3 for *w* = 0.1 we have *φ* ≃ 0.4, so that the activity coefficient 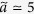. Figure 3 also shows the concentration and temperature dependence of the interfacial tension^24^ parameters 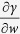 and 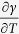, the latter using the normalization implied by eq. (17).

The mass transfer coefficient *k*_g_ is the only unknown parameter in the model. A common approach^19^ to estimate mass transfer coefficients is using correlations in terms of a dimensionless group known as the Sherwood number: S*h* = *k*_g_*h*/*ρ*_g_*D*_g_, where *ρ*_g_ and *D*_g_ are the density and (ethanol) mass diffusivity, respectively, for the gas phase. The Sherwood number indicates the relative importance of convective and diffusive mass transfer, and for essentially stagnant fluids it is reasonable to set Sh ~ 1. Using the values 
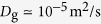
 and 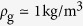 we obtain the following estimate for the mass transfer coefficient: 
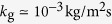
. We note that this value is consistent with measured evaporation rates in ethanol-water mixtures^14^.

Figure 6 shows the dependence of 〈*v*_*z*_〉_0_/*V* as a function of ethanol mass fraction *w*_0_ obtained from eq. (17). From this figure we see the velocity induced by a concentration gradient alone goes through a maximum, while the velocity induced by a temperature gradient alone increases monotonically with *w*_0_. The 〈*w*〉′ contribution to 〈*v*_*z*_〉_0_ scales roughly with 
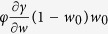
; the explicit dependence on *w*_0_ produces a maximum at *w*_0_ = 0.5. However, since both 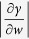 and *φ* are decreasing functions of *w*_0_ as shown in Fig. 3, the maximum in the 〈*w*〉′ curve is shifted to 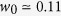. For 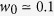, the velocity induced by a temperature gradient 〈*T*〉′ is roughly one-third of that induced by a concentration gradient 〈*w*〉′, while for 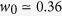 the individual contributions are equal. The velocity induced by the combined effects of concentration and temperature gradients shown in Fig. 6 goes through a maximum at the ethanol concentration for a typical wine. Similar calculations that include water evaporation differ by roughly 10% from the curves in shown Fig. 6. It is important to note that both the shapes and relative magnitudes of the curves in Fig. 6 are independent of the estimated model parameter *k*_g_, and instead are determined by the simple physical model and thermodynamic properties of ethanol-water mixtures shown in Fig. 3.

We have also made infrared thermography measurements on cognac (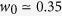), which are presented in Fig. 7. As for the wine film in Fig. 4, we see in Fig. 7 two cooler regions (falling tears evident from video in Supplementary Information) surrounding a region with a temperature gradient. The temperature profile in Fig. 8 shows a region with nearly uniform temperature for which we presently do not have an explanation. A rough estimate of the average temperature gradient from the profile in Fig. 8 gives *T*′ ~ −200 K/m, which leads to a Marangoni stress of 
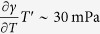
. We estimate the concentration gradient using (16), which gives *w*′ ~ −0.6 1/m and a Marangoni stress of 
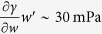
. Hence, for the case of a liquid with higher ethanol concentration, we find that the relative contribution of the temperature gradient to the Marangoni stress is approximately the same as that from concentration gradient, and the combined contributions lead to a slightly smaller Marangoni stress as that for the lower ethanol content liquid. These observations are consistent with the results in Fig. 6. It is worth noting that both the value and concentration independence of *w*′ found in this study are consistent with measured concentration profiles in evaporating ethanol-water films^9^. We also note that previous experimental work, where thermal effects have been ignored, has been based on ethanol-water mixtures having higher ethanol concentrations *w* ≥ 0.5^9^,^14^.

We now consider the mechanisms responsible for the formation of wine tears. As shown in Fig. 1, tears are formed in the ridge near the top of the film. A simple explanation for the formation of the ridge^11^,^12^,^13^,^14^ is that the flow rate induced by Marangoni stresses exceeds the rate at which the volume of the film increases by the motion of the contact line. This implies the contact angle *α* will be larger in an evaporating liquid film than at equilibrium. A force balance at a stationary contact line, in the absence of mass transfer and viscous stresses, leads to Young’s equation: cos *α* = Δ*γ*^fs^/*γ*, where Δ*γ*^fs^ is the difference in interfacial tensions between the fluid phases and the solid^21^. Since the liquid at the ridge has a larger interfacial tension (less ethanol), Young’s equation assuming Δ*γ*^fs^ is constant suggests an increase in *α*. We have also observed a gradual increase in the measured contact angle during ethanol ethanol evaporation. Based on these observations, we assume for the contact angle: *α* ≃ 30° in the analysis that follows.

**Figure 2:**
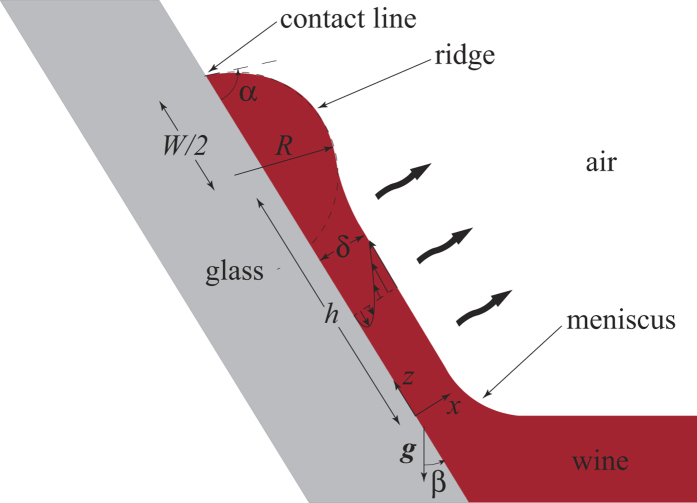
Schematic cross-sectional view of an evaporating liquid film (wine) on a solid surface (glass) showing a sketch of the velocity distribution given ineq. (6). Note that the film thickness *δ* is exaggerated for clarity.

**Figure 3:**
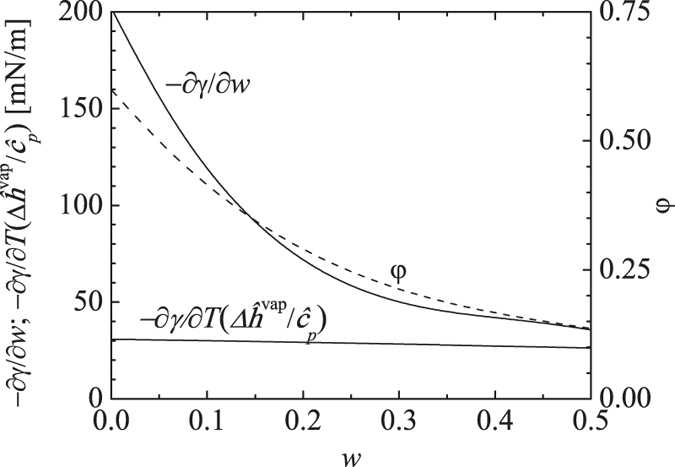
Interfacial tension derivatives −∂*γ*/∂*w* and −∂*γ*/∂*T* from data in^24^ where 
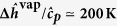
 (solid curves) and vapor-liquid equilibrium factor 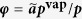 (dashed curve) where 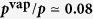 as functions of ethanol mass fraction at 298 K.

Studies on the dynamics and stability of free-surface flows date back to the mid-19^th^ century^27^ and continue to be an active area of research^28^,^29^. As noted above, there has been interest in understanding the flow instabilities that are observed in films driven by Marangoni stresses. Much of this work has focused on the formation of regularly spaced ridges in the meniscus region that are parallel to the *z*-direction of Fig. 2. This instability appears to be driven by a competition between viscous, capillary and Marangoni stresses^11^,^12^,^13^,^14^. We have not observed this phenomenon, presumably because the spacing of the ridges decreases as the film becomes more vertical (smaller *β*)^9^.

It has been suggested^11^ that the formation of tears from the ridge is the result of the well-known Rayleigh-Plateau instability^27^. The Rayleigh-Plateau instability, which describes the formation droplets from a liquid jet, is based on the interplay between inertial and interfacial tension forces that result from axisymmetric disturbances to the surface of the liquid jet. Rayleigh found that a liquid jet is unstable to disturbances having a wavelength *λ* larger than the circumference of the cylinder 2*πR*. The hypothesis that this mechanism is responsible for wine tears was not, however, quantitatively investigated^11^.

To investigate the instability leading to wine tear formation, we begin with a determination of the morphology of the ridge. A crude approximation is to treat the ridge as a cylinder with radius *R* (see Fig. 2). An estimate for *R* can be obtained from the width of the ridge *W* and contact angle: *W* = 2 *R* sin*α*. An analysis of images (like those shown in Fig. 1) gives 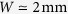, so that 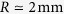. From these images we also estimate the average spacing between the falling tears to be 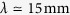. The relative importance of viscous and interfacial forces in liquid film dynamics can be ascertained from the value of the Ohnesorge number 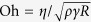. Here, we have Oh ~ 10^−3^, so it is reasonable to assume viscous effects do not play a role in the instability mechanism. The fastest growing instability for the Rayleigh-Plateau instability corresponds to 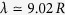
27. Hence, the predicted wavelength for the Rayleigh-Plateau instability is 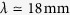, which is in good agreement with the observed value. A somewhat more realistic morphology for the ridge is to treat it as a cylinder that has been cut along its length and is bound by two contact lines. The stability of liquid ridges having this geometry have been investigated; the most unstable mode corresponds to a disturbance having wavelength 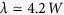
30,31. For the system considered here, this gives 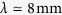, which is roughly a factor of two smaller than the observed value, but still reasonable. Based on this analysis, we believe there is strong evidence that *the regular pattern of wine tear formation is due to the Rayleigh-Plateau instability*.

## Discussion

The tears of wine phenomenon is the result of a delicate interplay between interfacial and bulk hydrodynamics. The evaporation of ethanol induces an interfacial (Marangoni) stress that in turn induces an observable bulk flow. A common misconception is that the Marangoni stress arises because of concentration gradients alone. We have shown, using a combination of experiments and modeling, that the Marangoni flow taking place in the tears of wine phenomenon is the result of both composition and temperature gradients. Infrared thermography measurements reveal the existence of temperature gradients of sufficient magnitude to induce a Marangoni stress comparable to that from concentration gradients.

The model developed here represents a simple description of the coupling of fluid flow and energy and mass transport in evaporating liquid films. In contrast to previous analysis of the phenomenon in which the interfacial stress was treated as a parameter, the model developed here is based on a coupled set of balance equations for mass, momentum and energy so that the interfacial stress is predicted. The model does not take into account more complex phenomena in regions of the film near the meniscus and contact line, and is only able to capture qualitative features of measured temperature profiles. In particular, an explanation for the non-monotonic dependence of temperature observed is some cases requires further investigation. The evaporation of water, which was neglected in this work, further complicates the phenomenon. The large latent heat of vaporization of water means that the thermal effect is enhanced, while at the same time water evaporation will decrease the concentration contribution to the Marangoni stress. In addition, we have neglected coupling of diffusive mass and energy fluxes (Soret and Dufour effects) and the enthalpy of mixing. Nevertheless, the model semi-quantitatively predicts the conditions necessary for the observation of the tears of wine and establishes the essential nature of thermal effects in this phenomenon. The dependence of the Marangoni stress on ethanol concentration is strongly influenced by the thermodynamic properties (interfacial tension and activity coefficient) of ethanol-water mixtures. Interestingly, the combination of these properties results in a maximum Marangoni stress at the ethanol concentration found in a typical wine.

A second interesting feature of the tears of wine phenomenon is the highly-regular pattern in which the tears form. Using a rather simple analysis based on a simplified morphology for the wine film, we have provided strong evidence that the pattern observed in wine tear formation is the result of the well-known Rayleigh-Plateau instability.

## Methods

### Materials

The red wine (California Pinot Noir) used in this study was 13% ethanol by volume, which neglecting the volume change of mixing, corresponds to 10% ethanol by mass, and the cognac (Hennessy) contained 40% ethanol by volume, which corresponds to 35% ethanol by mass.

### Procedures

**Figure 4:**
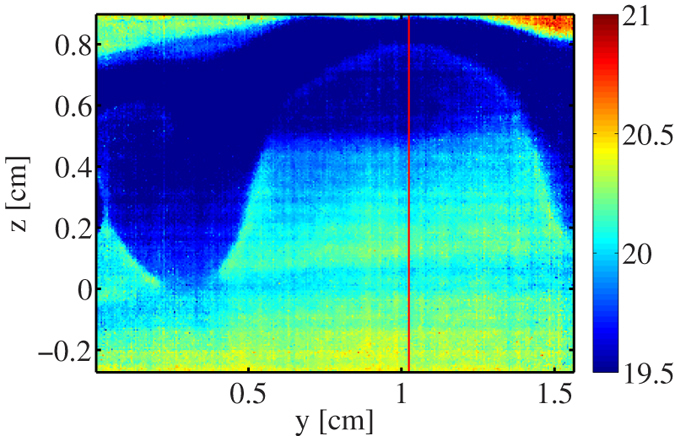
Infrared image of wine film (*w* ≃ 0.1) taken along the *x*-direction as defined in Fig. 2 Scale on right gives temperature in °C.

**Figure 5:**
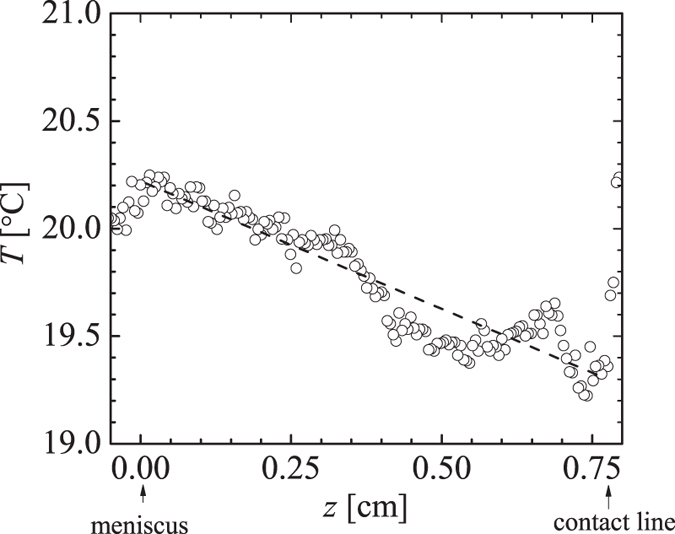
Temperature profile taken along the red line in Fig. 4. The dashed line has a slope of *T*′ ~ −100 K/m, which is the average value obtained from multiple images.

**Figure 6:**
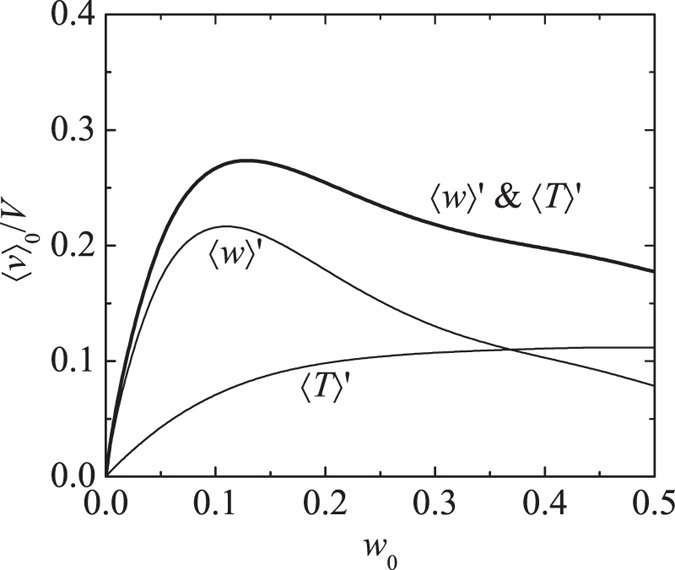
Average film velocity normalized by 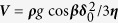 as a function of ethanol mass fraction as determined by eq. 17. Curves show velocity driven by a composition gradient 〈*w*〉′, temperature gradient 〈*T*〉′ and both composition and temperature gradients.

**Figure 7:**
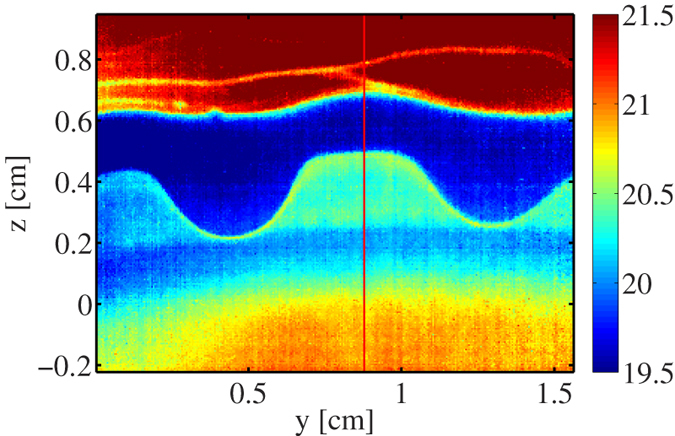
Infrared image of cognac film (*w* ≃ 0.35) taken along the *x*-direction as defined in Fig. 2 Scale on right gives temperature in °C.

**Figure 8:**
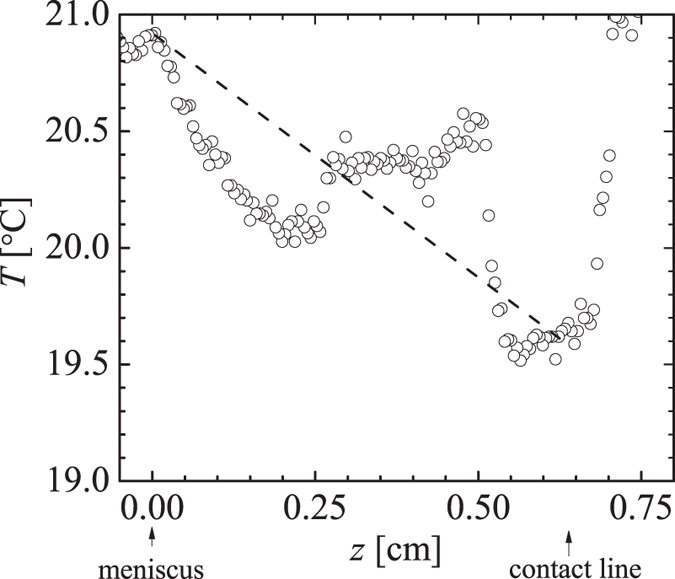
Temperature profile taken along the red line in Fig. 7 The dashed line has a slope of *T*′ ~ −200 K/m, which is the average value obtained from multiple images.

The glass was cleaned by soaking in a solution of chromic acid and hydrogen peroxide followed by rinsing with deionized water. The image in Fig. 1 is taken from a movie made with a reflected light camera (Sony DCRSR64) and modified lens to enhance the camera focus deep and to avoid glass wall reflection^4^. Infrared images in Figs 4 and 7 were obtained from a video obtained using an IR Camera (FLIR A320) having a spatial resolution of 320 × 240 pixels and sensitivity of 0.1 K equipped with an 18 mm focal length lens. The infrared movies were made in a glass with a conical shape (*β* ≃ 45°) to facilitate imaging of the liquid film. The contact angle of wine on (borosilicate) glass was determined using reflected light differential interferometry by placing a 2 − 3 *μ*l drop on a microscope slide. Details of the method can be found elsewhere^32^,^33^.

## Additional Information

**How to cite this article**: Venerus, D. C. and Nieto Simavilla, D. Tears of wine: new insights on an old phenomenon. *Sci. Rep.* **5**, 16162; doi: 10.1038/srep16162 (2015).

## Supplementary Material

Supplementary Information

Supplementary Video 1

Supplementary Video 2
