# Supplementary material for: Tears of wine: new insights on an old phenomenon
Source: Sci Rep. 2015 Nov 9;5:16162. doi: 10.1038/srep16162 (PMC4637898; doi:10.1038/srep16162)
Supplement: Supplementary Information [file srep16162-s1.pdf]

Supplementary Information

Tears of wine: new insights on an old phenomenon

David C. Venerus and David Nieto Simavilla

Department of Chemical and Biological Engineering

Illinois Institute of Technology, Chicago, IL 60616

Two infrared videos of tears of wine phenomenon:

1. Infrared thermographic video of the meniscus region of a glass containing red wine. The video was obtained using an IR Camera (FLIR A320) having a spatial resolution of 320 x 240 pixels and sensitivity of 0.1 K at ten frames per second equipped with an 18 mm focal length lens. (IRTmoviewine.mov)

2. Infrared thermographic video of the meniscus region of a glass containing cognac. The video was obtained using an IR Camera (FLIR A320) having a spatial resolution of 320 x 240 pixels and sensitivity of 0.1 K at ten frames per second equipped with an 18 mm focal length lens. (IRTmoviecognac.mov)
